# Does Mental Health First Aid training improve the mental health of aid recipients? The training for parents of teenagers randomised controlled trial

**DOI:** 10.1186/s12888-019-2085-8

**Published:** 2019-03-27

**Authors:** Amy J. Morgan, Julie-Anne A. Fischer, Laura M. Hart, Claire M. Kelly, Betty A. Kitchener, Nicola J. Reavley, Marie B. H. Yap, Stefan Cvetkovski, Anthony F. Jorm

**Affiliations:** 1 Centre for Mental Health, Melbourne School of Population and Global Health, University of Melbourne, Melbourne, Victoria 3010 Australia; 2 Mental Health First Aid Australia, Parkville, Australia; 3 School of Psychology and Public Health, La Trobe University, Melbourne, Australia; 4 School of Psychological Sciences, Monash Institute of Cognitive and Clinical Neurosciences, Monash University, Clayton, Australia; 5 Centre for Urban Research, RMIT University, Melbourne, Australia; 6 Faculty of Health, School of Psychology, Deakin University, Burwood, Australia

**Keywords:** Mental health first aid, Social support, Help-seeking behavior, Mental disorders, Adolescent

## Abstract

**Background:**

There is well-established evidence that Mental Health First Aid (MHFA) training improves knowledge about how to support someone developing a mental health problem, but less evidence that this support improves the mental health of the recipient of aid. This randomised controlled trial aimed to assess the long-term effects of MHFA training of parents on the mental health of their adolescent children.

**Methods:**

384 Australian parents of an adolescent aged 12–15 were randomised to receive either the 14-h Youth MHFA course or the 15-h Australian Red Cross Provide First Aid course. Outcomes were assessed at baseline, 1-year, and 2-year follow-up in both parents and adolescents. Primary outcomes were cases of adolescent mental health problems, and parental support towards their adolescent if they developed a mental health problem, rated by the parent and adolescent. Secondary outcomes included parent knowledge about mental health problems, intentions and confidence in supporting a young person, stigmatizing attitudes, and help-seeking for mental health problems.

**Results:**

Parent and adolescent reports showed no significant difference between training groups in the proportion of cases of adolescents with a mental health problem over time (ps > .05). There was also no significant difference between training groups in the quality of parental support provided to their adolescent at 1- or 2-year follow-up (ps > .05). In contrast, some secondary outcomes showed benefits from the Youth MHFA training relative to the control, with increased parental knowledge about mental health problems at 1-year (d = 0.43) and 2-year follow-up (d = 0.26), and increased confidence to help a young person (d = 0.26) and intentions to provide effective support (d = 0.22) at 1-year follow-up.

**Conclusions:**

The study showed some improvements in mental health literacy in training recipients, but could not detect changes in the mental health of adolescents and the support provided to them by their parents if they had a mental health problem. However, there was a lack of power to detect primary outcome effects and therefore the question of whether MHFA training leads to better outcomes in the recipients of aid remains to be further explored.

**Trial registration:**

ACTRN12612000390886, registered retrospectively 5/4/2012.

**Electronic supplementary material:**

The online version of this article (10.1186/s12888-019-2085-8) contains supplementary material, which is available to authorized users.

## Background

Mental disorders often have their first onset during adolescence [1]. However, many young people with mental disorders do not receive professional help, or there is a substantial delay in seeking help [2, 3]. Barriers to help-seeking include a lack of knowledge about mental disorders and which professional help-seeking options are available [4], embarrassment or concern about what others might think, and a preference to seek help from family rather than professionals [5, 6]. As such, parents are ideally placed and motivated to recognise when their child may be developing a mental health problem, respond in a supportive manner and facilitate early professional help-seeking [7]. In order to provide this support, parents themselves need the relevant knowledge and skills. However, surveys of the Australian public show that many adults also have limited mental health literacy [4].

An intervention which has the potential to improve the support that parents provide to adolescents developing a mental disorder is Mental Health First Aid (MHFA) training. MHFA training is a course for members of the public in how to assist someone who is developing a mental illness or experiencing a mental health crisis situation (e.g. the person is suicidal or has had a traumatic experience). This first aid is given until the person receives appropriate professional help or until the crisis resolves. The course teaches adults how to give mental health first aid using a five-component MHFA Action Plan: Approach the person, assess and assist with any crisis; Listen non-judgmentally; Give support and information; Encourage the person to get appropriate professional help; and Encourage other supports [8]. MHFA training began in Australia in 2000 and has expanded rapidly. MHFA Australia supports over 1500 accredited instructors who have trained over 700,000 adults. Furthermore, the program has spread to 25 other countries. As well as the Standard MHFA course for adults, there is a tailored course for adults assisting adolescents (Youth MHFA), which contains additional teaching about eating disorders, non-suicidal self-injury, adolescent development and effective communication with an adolescent.

There has been extensive research on the effects of MHFA training. A recent meta-analysis of 18 controlled trials [9] found benefits up to 6 months after training, with improved mental health first aid knowledge (d = 0.54), recognition of mental disorders (d = 0.52), beliefs about effective treatments (d = 0.19), and stigmatizing attitudes (d = 0.14). Improvements were also observed in confidence to help a person with a mental health problem (d = 0.46), intentions to provide first aid (d = 0.55), and the amount of help provided to a person with a mental health problem (d = 0.23). However, few studies examined the quality of help that was offered and the impact on the recipient’s mental health. Hence, there is well-established evidence that MHFA training improves mental health literacy and how to support someone developing a mental health problem, but less evidence that this support improves the mental health of the recipient of the aid. In most studies, the recipients cannot be contacted and benefits must be inferred from reports of the first aiders. Only two controlled trials have examined the mental health of aid recipients [10, 11], showing a small non-significant improvement up to 6 months after training (d = 0.14, 95% CI -0.05 to 0.33). The meta-analysis also found that the vast majority of evaluations have been on the standard adult course, with few controlled trials evaluating the Youth MHFA course [10, 12]. Furthermore, there was limited research on whether training benefits lasted longer than 6 months.

The present study aimed to evaluate the 14-h Youth MHFA training course with parents of young adolescents to examine (a) whether the training helps parents to provide better support if their adolescent develops a mental health problem, and (b) whether this support benefits the adolescent’s mental health. The effects of the training were examined up to 2 years later, allowing the long-term impact of MHFA training to be assessed for the first time.

## Methods

### Study design

The study was a single-blind, parallel group superiority RCT with participants randomised to Youth MHFA (YMHFA) or Red Cross Provide First Aid (PFA) training in a 1:1 ratio. The trial was registered with the Australian and New Zealand Clinical Trials Registry (ACTRN12612000390886). Provide First Aid training was chosen as the control condition as it teaches parents useful skills but was not expected to have any mental health benefit. Furthermore, it controls for non-specific training effects and allows for the long-term impact of the intervention to be examined.

### Participants

Australian parents of an adolescent aged between 12 and 15 years were eligible to participate. One parent and one teenager per family could take part - registration was done as a dyad. Participants who had undertaken Standard or Youth MHFA or PFA training within the previous three years were ineligible to participate. The study was promoted to parents via newsletters and online communications in secondary schools, ads placed with community organisations (e.g. local libraries, sport centres) and community radio, and links from websites including a study Facebook page. Potential participants were directed to the trial website at http://www.tpot.net.au, where they could read about the study and consent to participate. Demographic data were collected during online registration, including a preferred time/day for telephone interview. Participating adolescents provided verbal assent at each interview. Recruitment took place between October 2011 and March 2016.

### Randomisation

Randomisation occurred during online registration using a random integer generator programmed on the trial website to give values of 1 = YMHFA and 2 = PFA. The program ran immediately after parents selected two potential training dates. Parents were then emailed an electronic copy of their consent form and selected course dates, and informed that they would be contacted in 2–3 weeks to complete their baseline interview. Course allocation was only revealed at the conclusion of the parent interview. Although blinding of parent participants to intervention was not possible, interviewers carrying out the assessments were blinded.

### Interventions

The two training courses were delivered by accredited YMHFA or PFA instructors to groups of parents ranging in size from approximately 5–18. Adolescents did not receive any direct intervention. YMHFA was taught across 4 × 3.5 h sessions, usually run over two consecutive days, while PFA was taught over 2 sessions. Parents were given up to four months to attend their allocated course after completing the baseline interview. In July 2014 parents were sent a $50 gift card after course attendance to reimburse travel expenses. This initiative was extended to parents who previously attended their course and remained in the study. Training venues included those currently used by instructors for publicly run YMHFA courses or PFA training (e.g. Red Cross training centres, community centres or school halls). Both control and intervention courses were timetabled to coincide at the same venue in different rooms where possible, or within the same geographical area but at different venues, at similar times. This was to maintain consistency of the course setting while preventing contamination. Both courses included a manual for participants [13, 14].

#### Youth mental health first aid course

The 14-h YMHFA training teaches adults, who care for or work with adolescents (those aged between 12 and 18 years), the skills needed to:

- recognise the early signs of a mental health problem, e.g. depression, anxiety, psychosis, substance use problems or eating disorders;
- identify potential mental health-related crises, e.g. suicidal thoughts and behaviours, non-suicidal self-injury (sometimes called deliberate self-harm), panic attacks, traumatic events, severe psychotic states, acute effects of drug or alcohol use and aggressive behaviours, and
- assist adolescents to get appropriate professional help as early as possible.

A research assistant (JF) performed a fidelity content check for courses conducted by two YMHFA instructors. Each content section was scored 0–2 for not completed, partially completed, or fully completed. Instructor scores showed high fidelity to the course content (instructor 1 54/58, instructor 2 50/58).

### Red cross provide first aid course

The 15-h PFA training (HLTAID003) teaches the knowledge and skills to sustain life, reduce pain and minimise the consequences of injury and illness until professional help arrives. It covers a range of topics, including cardiopulmonary resuscitation, drowning, anaphylaxis, airway obstruction, bleeding and wound care, head, neck and spinal injuries, poisoning, envenomation, seizure management, stroke and unconsciousness.

### Outcomes

Outcomes were measured using computer-assisted telephone interviews carried out by a telephone survey company. Interviews were conducted with both the parent and adolescent independently at baseline, 1 year after baseline and 2 years after baseline. All parents were posted a primary approach letter 1–2 weeks prior to their scheduled follow-up interview call, and up to 10 attempts were made to schedule and complete the telephone interviews.

#### Primary outcomes

##### Adolescent mental health

This was assessed by the 25-item Strengths and Difficulties Questionnaire (SDQ) [15] Parent and Child Report versions, adapted for phone interview. Likely cases of mental disorders were identified by the total difficulties scores in the abnormal range of 17 or greater for parent report, and 20 or greater for adolescent self-report [16, 17]. Internal consistency of total difficulty scores was excellent, ω = .88 (parent) and ω = .86 (adolescent).

##### Perceptions of parent support by adolescents with a mental health problem

This was assessed by asking adolescents a series of questions: (1) “Over the last 12 months, have you had any sort of mental health problem?” If yes, (2) “what do you think the problem was?” (3) “Did you get help from family?”, and (4) “How well did your mother/father support you when you had your mental health problem?” (asked about each adolescent’s parent, as appropriate). Responses were “Very well”, “Fairly well”, “Not well”, or “Unsure”. If the adolescent was unsure what was meant by a “mental health problem”, they were told “a period of weeks or more when you are feeling depressed, anxious, emotionally stressed, or are misusing alcohol or drugs, and these problems are interfering with your life”. Responses were dichotomised into (1) “Very well” versus (0) “Fairly well”, “Not well”, or “Unsure”.

##### Quality of parental support towards adolescents with a mental health problem

This was assessed by questions to the parent adapted from a previous survey [18]. (1) “Over the last 12 months has [your child] had any sort of mental health problem?” If yes, (2) “what do you think the problem was?”, (3) “Over the last 12 months, have you done anything to help him/her with this mental health problem?”, (4) “What did you do?”. Open-ended responses to actions taken were scored blinded to allocation and measurement occasion, according to congruence with the MHFA Action Plan. [8] Responses received a score of 0–2 points for each component of the plan: Approach the person, assess and assist with any crisis; Listen non-judgmentally; Give support and information; Encourage the person to get appropriate professional help; and Encourage other supports. Total scores on the quality of actions taken could range from 0 to 12. A random sample of 50 responses was double-coded and inter-rater reliability (ICC) was 0.83 (95% CI 0.60 to 0.92).

##### Secondary outcomes

A variety of secondary outcomes were included in the trial (see Table 1 for a description of each). Some of these were in response to 4 hypothetical vignettes describing an adolescent with a mental health problem: depression, social phobia, an eating disorder not otherwise specified (e.g. EDNOS or OSFED) and psychosis (see Additional file 1 for the vignettes). Secondary outcomes assessed various aspects of parent mental health literacy that would be expected to improve after MHFA training, including recognition of mental health problems, knowledge about youth mental health problems, confidence and intentions to help a person with a mental health problem, stigma towards people with mental health problems, and appropriate help-seeking for mental health problems. In adolescents, secondary outcomes included perceived general social support from parents, intended and actual help-seeking from parents for a mental health problem, help-seeking from a health professional, and stigmatising attitudes. Stigma was assessed in adolescents to examine whether improvements in parent stigma influenced their children’s attitudes.

**Table 1:** Overview of secondary outcomes

Outcome	Survey measure	Response	Scoring	Psychometrics
Outcomes completed by both parents and adolescents				
Social Distance [26]	5 questions assessing desire for social distance from the person in the psychosis vignette. E.g. *Would you be happy for your child / Would you be happy…to develop a close friendship with X?*	4-point Likert scale 1 = Yes definitely 2 = Yes, probably 3 = Probably not 4 = Definitely not	Mean score of 5 questions, range 1–4.	Parent ω = .90 Adolescent ω = .88
Stigma: Weak-not-sick [26]	4 questions assessing that the person in the psychosis vignette is weak not sick. E.g. *X could snap out of it if (he/she) wanted*	5-point Likert scale 1 = Strongly disagree 2 = Disagree 3 = Neither agree nor disagree 4 = Agree 5 = Strongly agree	Mean score of 4 questions, range 1–5, dichotomised to low stigma (=1) or not (> 1).	Parent ω = .81 Adolescent ω = .56
Stigma: Dangerous/Unpredictable [26]	4 questions^a^ assessing that the person in the psychosis vignette is dangerous or unpredictable. E.g. *X’s problem makes (him/her) unpredictable*	5-point Likert scale 1 = Strongly disagree 2 = Disagree 3 = Neither agree nor disagree 4 = Agree 5 = Strongly agree	Mean score of 4 questions, range 1–5.	Parent ω = .46 Adolescent ω = .45
K6 psychological distress (12-month version) [27]	6 questions assessing the frequency of psychological distress in the worst month during the past 12 months	5-point Likert scale 1 = None of the time 2 = A little of the time 3 = Some of the time 4 = Most of the time 5 = All of the time	Total scores of 19 or above indicate a high level of psychological distress	Parent ω = .91 Adolescent ω = .93
Outcomes completed by parents only				
Problem recognition	*What, if anything, do you think is wrong with X?* Question in response to each of 4 vignettes: depression, social phobia, EDNOS, psychosis	Verbatim responses were scored for correct/incorrect, blinded to group allocation and timepoint.	1 point for correct recognition if the following were mentioned: Depression (depression, depressed), social phobia (anxiety/anxious, social anxiety, social phobia, anxiety disorder, performance anxiety), EDNOS (Eating disorder, EDNOS, bulimia/bulimic/bulimia nervosa/mia, binge eating/loss of control eating, binge eating disorder, anorexia/anorexia nervosa/anorexic/ana, disordered eating), psychosis (schizophrenia/paranoid schizophrenia, psychosis/psychotic, schizophrenic, schizoaffective disorder, precursor to schizophrenia). Mean score of 4 responses, range 0–1.	ω = 0.82
Quality of MHFA intentions	*Imagine X is your child. You want to help (him/her). What would you do?* Question in response to each of 4 vignettes: depression, social phobia, EDNOS, psychosis	Verbatim responses were scored for consistency with the ALGEE action plan in the Youth MHFA manual, [8] blinded to group allocation and timepoint.	Mean score of 4 responses, range 0–12.	Inter-rater reliability (ICC) for 4 vignettes ranged from 0.74–0.84. ω = 0.56
Confidence to help	*How confident would you be in your ability to help X?* Question in response to each of 4 vignettes: depression, social phobia, EDNOS, psychosis	4-point Likert scale 1 = Very confident 2 = Fairly confident 3 = Slightly confident 4 = Not confident at all	Mean score of 4 responses, range 1–4.	ω = 0.84
Knowledge about mental health problems	18 questions on knowledge about mental health problems, derived from the Youth MHFA manual. [8] E.g. *If a teenager has a traumatic experience, it is best to make them talk about it as soon as possible*	Agree, Disagree, Don’t know	1 point for a correct response to each question, range 0–18.	Test-retest r = 0.67 (PFA group)
Quality of MHFA support towards other person	*Over the last 12 months, has anyone else you know had any sort of mental health problem?...Have you done anything to help the person with this problem?...What did you do?*	Verbatim responses were scored for consistency with the ALGEE action plan, blinded to group allocation and timepoint.	Scores range 0–12.	Inter-rater reliability ICC = 0.76
Appropriate help-seeking for mental health problem	*Over the last 12 months, have you yourself had any sort of mental health problem?...Have you done anything to deal with this mental health problem?...What did you do?*	Verbatim responses were scored for the presence of an appropriate source of help.	Scored a 1 if mentioned any of the following professionals or treatments: taking medication, antidepressant, anxiolytic, counselling, counsellor, GP, psychologist, psychiatrist, CBT, hospital, other medical (mental health practitioner or specialist, professional for alcohol misuse, maternal & child health nurse), professional organisation (Centre Against Sexual Assault, Drummond Family Services).	
Outcomes completed by adolescents only				
Perceived general social support from parent [28]	Communication subscale of Parent Attachment from the People in My Life Questionnaire (5 questions). E.g. *My parents can tell when I am upset about something.*	4-point Likert scale 1 = Almost never or never true 2 = Sometimes true 3 = Often true 4 = Almost always or always true	Scores range 5–20.	ω = 0.82
Intended help-seeking from parent	*If you had a problem right now like X would you ask for help?...Where would you go?* Question in response to each of 4 vignettes: depression, social phobia, EDNOS, psychosis	Verbatim responses.	Scored 1 point per vignette if mentioned seeking help from a parent. Sum score of 4 responses, range 0–4.	ω = 0.71
Actual help-seeking from parent	*Over the last 12 months, have you had any sort of mental health problem?... Did you get help from family?... Who in the family?*	Verbatim responses.	Scored a 1 if adolescent reported seeking help from either or both parents.	
Help-seeking from a health professional	*Over the last 12 months, have you had any sort of mental health problem?... Did you get help from a health professional or counsellor?*	Verbatim responses.	Scored a 1 if adolescent responded with yes.	

Note: ω was calculated as Revelle’s omega total for total scores

^a^3 questions only for adolescents, see Yap et al. 2014 [26]

### Sample size estimation

The required sample size was based on the primary outcome of perceptions of parent support by adolescents who have developed a mental health problem, estimated based on two statistics: (1) the proportion of adolescents who would develop a mental health problem by the first follow-up and (2) the size of the effect of the intervention on perceived support in that sub-group. Based on an analysis of data on past 12-month prevalence of reported depression symptoms in 12–17-year olds in the 2006 National Survey of Youth Mental Health Literacy [5], and past 12-month prevalence of anxiety and/or depressive disorders in 16–18 year olds [19], we estimated that the 12-month prevalence of mental health problems (anxiety and/or depression symptoms) for the adolescents in this study would be 15.5%. A medium effect size was chosen to evaluate the difference between adolescents who developed a mental health problem, as a smaller effect size may not be practically meaningful. According to Stata Release10, for a repeated measures design, with the change method of sample size calculation and assuming a 0.60 correlation between pre and follow-up measurements, to detect an effect size of d = 0.44, with a power = 0.80 and an alpha = 0.05, 64 adolescents with a perceived mental health problem would be needed per intervention and control group. To ensure a sufficient sample size to detect the difference between the sub-group of adolescents with a perceived mental health problem, we required a full sample of 413 per group. Increasing the sample size by 20% to account for attrition, the total sample size that was required was 990 adolescent-parent dyads (495 per intervention and control group).

### Adverse events

There were no harms reported in this trial. Participants were provided with phone numbers for several services offering free 24-h telephone crisis or counselling support (e.g. Lifeline) in the event they felt distressed during the training or phone interviews and were encouraged to advise the trial manager if they used these resources. Participants were instructed to alert the telephone interviewer if they were feeling distressed during the phone surveys, and their course instructor if they felt distressed during the course. Adolescents aged 18 or older who were asked questions about suicide as part of the telephone interview were offered a postcard with information on suicide prevention. However, none were requested.

### Ethics

The study was approved by the University of Melbourne Human Research Ethics Committee.

### Statistical analysis

Data were analysed according to the intention-to-treat principle, using mixed-effects models for continuous and binary outcome variables, with group by measurement occasion interactions. This method of analysis is well suited to the data as it takes into account the hierarchical structure of the data, i.e. the correlation of measurement occasions within participants. These maximum likelihood-based methods are able to produce unbiased estimates when a proportion of the participants withdraw before the completion of the study, based on the reasonable assumption that these data are missing at random [20]. To help meet the missing at random assumption, parent age and education were included as fixed effects in models analysing parent outcomes, and adolescent SDQ caseness was included in models analysing adolescent outcomes. For continuous outcome measures with no substantial baseline imbalance, effect sizes (Cohen’s d) were calculated by dividing the difference between the two group means at follow-up by their pooled standard deviation. With baseline imbalances, Cohen’s d was calculated by dividing the mean change in each condition by the pooled standard deviation at follow-up. Analyses were performed in Stata 14 and RStudio and the significance level was set at *p* < .05.

## Results

### Participant flow and numbers analyzed

**Fig. 1:**
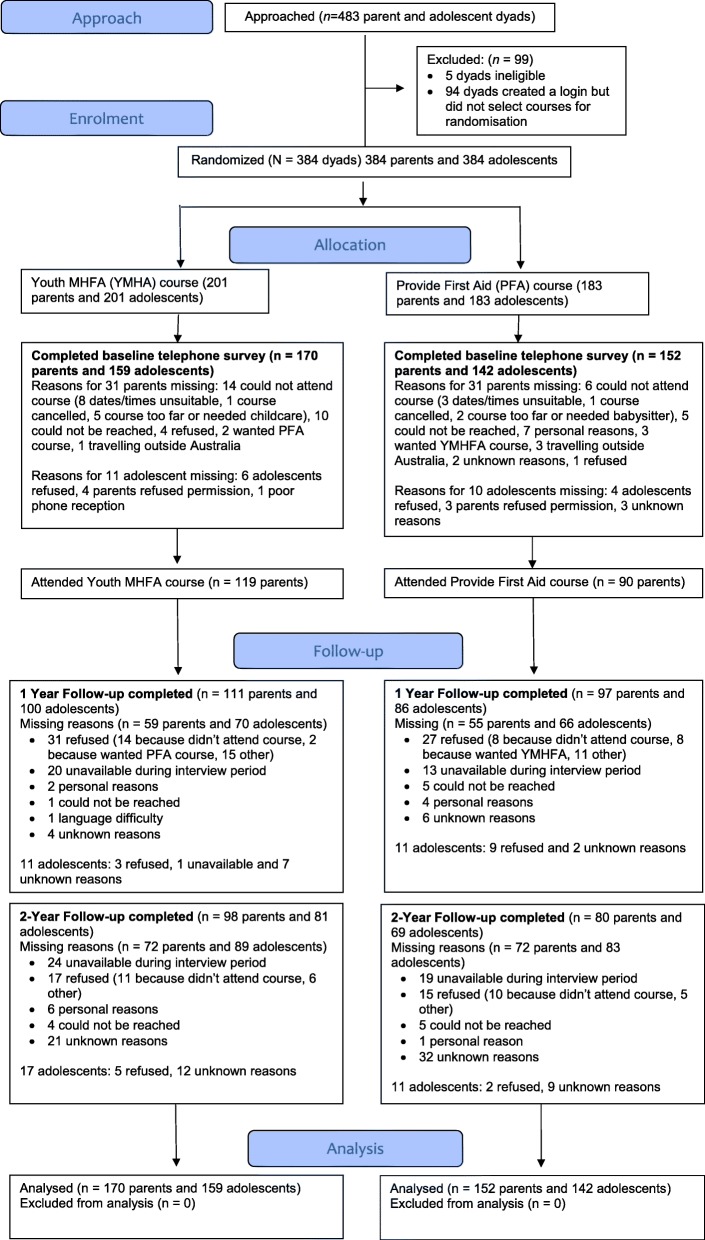
CONSORT flow diagram

Figure 1 shows the CONSORT flow diagram of the number of participants at each stage of the trial. All the participants included in the analyses completed the baseline questionnaire. Parent attrition rates were 35.4% at 1-year follow-up and 44.7% at 2-year follow-up. There were 219 parents (68.0%) with data at 1- or 2-year follow-up. Predictors of missingness at both follow-up assessments were explored with simple logistic regression models. Parents were less likely to be missing at follow-up if they had a tertiary education (OR = 0.58, 95% CI 0.36 to 0.93) or were younger in age (OR = 0.91, 95% CI 0.87 to 0.96). Adolescents were more likely to be missing at follow-up if they scored in the abnormal range on the SDQ at baseline (OR = 2.82, 95% CI 1.32 to 6.00).

### Participant characteristics

The two groups were similar in baseline sociodemographic characteristics, indicating that randomisation resulted in comparable groups (see Table 2). Participating families tended to consist of parents in a married or de facto relationship with approximately two children. Most participating parents were female with a mean age of 45.2 years. There were slightly more female adolescents than males, with a mean age of 13.3 years. Just over a quarter of parents indicated that their child had a health issue at baseline, most commonly anxiety (6.8%), asthma (6.5%), depression (3.7%) or ADHD (3.1%). Not all parents attended their respective training course, and course completion rates were lower for the PFA course than the YMHFA course (59.2% vs 70.0%, *p* = .043).

**Table 2:** Baseline participant characteristics

	YMHFA (*n* = 170)	PFA (*n* = 152)	p
Parent			
Family situation %			.675
Child living with both parents	71.8	75.7	
Parents separated but both involved in care of child	12.9	9.2	
Parents separated but only respondent involved in care of child	7.1	5.3	
Sole parent	6.5	6.6	
Other (e.g. grandparents)	1.8	3.3	
Married/defacto %	74.1	80.9	.146
Female %	89.4	86.8	.476
Tertiary education %	56.6	50.7	.294
Speaks only English at home %	85.9	79.6	.135
Not Aboriginal or Torres Strait Islander %	99.4	98.7	.209
Employed full or part time %	66.5	71.7	.310
Studying status %			.185
Full-time	2.9	3.9	
Part-time	16.5	11.2	
Not studying	11.2	18.4	
Unknown	69.4	66.4	
Age M (SD)	45.2 (5.54)	45.1 (5.69)	.867
Number of children M (SD)	2.2 (0.8)	2.3 (1.0)	.282
Child			
Female %	58.8	52.0	.217
Health issue %	26.5	27.0	.919
Age M (SD)	13.3 (1.11)	13.3 (1.08)	.949

Parents and adolescents were asked if the adolescent had developed a mental health problem in the past 12 months. At baseline, 60 adolescents (20.1%) reported a mental health problem, 32 (17.4%) at 1-year follow-up, and 32 (21.3%) at 2-year follow-up. Parents identified more adolescents with mental health problems: 93 (29.8%) at baseline, 52 (25.7%) at 1-year follow-up, and 54 (30.9%) at 2-year follow-up. Anxiety or depression were the most common problems reported by both. Reports of mental health problems showed moderate stability across time. Of 49 cases of mental health problems reported by adolescents across the follow-up period, 18 (36.7%) were present at baseline. For parents, of the 81 cases reported at 1 or 2 year follow-up, 40 (49.4%) were present at baseline.

### Primary outcomes

Participants’ scores on the SDQ indicated a minority of adolescents were likely to have a mental disorder at each timepoint, with higher proportions when rated by parents than adolescents (see Table 3). Rates of child-rated disorders in the MHFA group dropped by half from baseline to 2-year follow-up, whereas the rate in the PFA group was similar throughout. Despite this, there was no significant difference over time between groups in the proportion of adolescents likely to have a mental disorder based on parent or child-rated SDQ scores.

**Table 3:** Primary outcomes at baseline and follow-ups for Physical First Aid and Youth Mental Health First Aid groups

													Change over time between YMHFA and PFA	
	Baseline				1 year follow-up				2 year follow-up				Baseline to 1-year follow-up	Baseline to 2-year follow-up
	PFA		YMHFA		PFA		YMHFA		PFA		YMHFA			
	n/N	%	n/N	%	n/N	%	n/N	%	n/N	%	n/N	%	OR (95% CI)	OR (95% CI)
Adolescent with mental health problem – parent reported^a^	19/151	12.6	31/168	18.5	8/96	8.33	15/109	13.8	10/80	12.5	14/98	14.3	2.03 (0.23 to 17.54)	0.44 (0.05 to 4.14)
Adolescent with mental health problem – adolescent reported^b^	11/142	7.75	20/159	12.6	6/86	6.98	12/100	12.0	5/69	7.25	5/81	6.17	0.80 (0.12 to 5.33)	0.24 (0.02 to 2.26)
Mother supported adolescent very well^c^	14/26	53.9	17/30	56.7	6/10	60.0	8/22	36.4	2/11	18.2	8/20	40.0	0.21 (0.01 to 6.07)	6.55 (0.12 to 343.44)
Father supported adolescent very well^c^	7/23	30.4	7/29	24.1	3/8	37.5	2/21	9.52	2/10	20.0	5/19	26.3	0.34 (0.01 to 9.71)	3.65 (0.12 to 111.62)
	PFA (*n* = 38)		MHFA (*n* = 54)		PFA (*n* = 24)		MHFA (*n* = 28)		PFA (*n* = 18)		MHFA (*n* = 34)		Baseline to 1-year follow-up	Baseline to 2-year follow-up
	M	SD	M	SD	M	SD	M	SD	M	SD	M	SD	M_diff_ (95% CI), d (95% CI)	M_diff_ (95% CI), d (95%CI)
Quality of parental support towards adolescents with mental health problem	2.55	1.18	2.41	1.19	2.29	1.08	2.46	1.00	2.61	1.09	2.82	1.45	0.37 (− 0.42 to 1.15), 0.17 (− 0.38 to 0.71)	0.37 (− 0.44 to 1.18). 0.16 (− 0.41 to 0.73)

a Identified by total difficulties scores in the abnormal range of 17 or greater on the Strengths and Difficulties Questionnaire Parent Report

b Identified by total difficulties scores in the abnormal range of 20 or greater on the Strengths and Difficulties Questionnaire Child Report

c Versus “Fairly well”, “Not well”, or “Unsure” combined (reference category)

M = mean, SD = standard deviation

In adolescents reporting a mental health problem, perceived parental support showed no significant difference between groups over time (see Table 3). Whilst the small number of participants may have restricted statistical power, there was also no clear pattern of results favouring the MHFA group.

The quality of mental health first aid support that the parents offered to their adolescent with a mental health problem was generally fairly low, and showed no significant difference in change over time between groups (see Table 3). There was a very small, non-significant difference favouring the MHFA group at 1-year follow-up (d = 0.17, 95% CI: -0.38 to 0.71) and at 2-year follow-up (d = 0.16, 95% CI: -0.41 to 0.73).

### Secondary outcomes

Of the secondary outcomes completed by parents, the strongest effects were observed in improvements in knowledge about mental health problems (see Table 4). Compared to the control group, the MHFA group showed small-to-medium improvements at 1-year follow-up, and small improvements at 2-year follow-up. At 1-year follow-up there were also small improvements in confidence to help a young person with a mental health problem, and the quality of intended support parents would offer. These effects were smaller and no longer significant at 2-year follow-up. No other parent secondary outcomes showed improvements over time relative to the control group. Adolescent secondary outcomes showed no significant differences between groups over time, except for dangerous/unpredictable stigma and K6 psychological distress scores, both of which favoured the control group at 1-year follow-up.

**Table 4:** Secondary outcomes at baseline and follow-ups for Physical First Aid and Youth Mental Health First Aid groups

	Baseline						1 year follow-up						2 year follow-up						Change over time between MHFA and PFA	
	PFA			MHFA			PFA			MHFA			PFA			MHFA			Baseline to 1-year follow-up	Baseline to 2-year follow-up
Variable	n	M	SD	n	M	SD	n	M	SD	n	M	SD	n	M	SD	n	M	SD	M_diff_ (95% CI), d^#^ (95% CI)	M_diff_ (95% CI), d^#^ (95% CI)
MHFA knowledge	152	9.77	2.76	170	10.28	2.71	97	10.01	2.75	111	11.90	2.44	80	10.31	2.52	98	11.82	2.36	1.25 (0.62 to 1.87)^***^, 0.43 (0.15 to 0.70)	0.84 (0.17 to 1.51)^*^, 0.26 (−0.03 to 0.56)
Problem recognition	152	0.63	0.35	170	0.66	0.31	96	0.66	0.35	111	0.75	0.30	80	0.67	0.33	98	0.77	0.28	0.05 (−0.03 to 0.12), 0.11 (− 0.16 to 0.39)	0.03 (− 0.04 to 0.11), 0.12 (− 0.18 to 0.41)
Quality of MHFA intentions	152	2.24	0.62	170	2.34	0.73	96	2.20	0.77	111	2.57	0.83	80	2.34	0.81	98	2.66	0.90	0.23 (− 0.01 to 0.47)^*^, 0.22 (− 0.05 to 0.50)	0.20 (− 0.05 to 0.45), 0.15 (− 0.14 to 0.45)
Confidence	151	2.25	0.68	169	2.36	0.61	96	2.10	0.70	110	2.05	0.56	80	2.18	0.69	98	2.17	0.59	−0.17 (− 0.34 to -0.00)^*^, 0.26 (− 0.01 to 0.53)	−0.10 (− 0.28 to 0.07), 0.23 (− 0.06 to 0.53)
Quality of MHFA support towards other person	86	2.23	1.16	113	2.29	1.14	46	2.35	1.08	70	2.40	1.01	45	2.16	1.30	67	2.30	1.15	−0.02 (−0.52 to 0.48), − 0.08 (− 0.52 to 0.36)	0.06 (− 0.44 to 0.56), -0.02 (− 0.46 to 0.42)
Social distance (parent)	145	2.16	0.66	160	2.08	0.63	91	2.04	0.64	107	1.88	0.61	78	1.87	0.68	96	1.86	0.48	−0.11 (−0.26 to 0.04), 0.18 (− 0.11 to 0.46)	0.08 (− 0.08 to 0.24), −0.12 (− 0.43 to 0.19)
Dangerous/ unpredictable stigma (parent)	147	2.48	0.52	164	2.44	0.56	93	2.46	0.54	110	2.37	0.56	78	2.40	0.50	95	2.29	0.52	−0.04 (−0.20 to 0.12), 0.15 (− 0.12 to 0.43)	−0.06 (− 0.23 to 0.11), 0.22 (− 0.08 to 0.52)
Social distance (adolescent)	140	2.23	0.61	157	2.20	0.66	85	2.12	0.60	99	2.17	0.63	69	2.13	0.62	81	2.15	0.60	0.09 (−0.07 to 0.25), -0.08 (− 0.37 to 0.21)	−0.02 (− 0.19 to 0.15), 0.03 (− 0.35 to 0.29)
Weak not sick stigma (adolescent)	142	2.33	0.65	157	2.29	0.75	85	2.04	0.65	100	1.93	0.75	68	1.86	0.64	81	1.75	0.69	0.01 (−0.18 to 0.19), -0.07 (− 0.36 to 0.22)	0.04 (− 0.16 to 0.24), -0.18 (− 0.51 to 0.14)
Dangerous/ unpredictable stigma (adolescent)	133	2.63	0.67	148	2.56	0.67	80	2.44	0.72	96	2.54	0.59	67	2.46	0.58	80	2.50	0.54	0.19 (0.01 to 0.38)^*^, -0.36 (−0.67 to 0.05)	0.06 (− 0.14 to 0.26), -0.04 (− 0.37 to 0.30)
Perceived general social support from parent	142	16.26	3.11	159	15.74	3.50	86	16.03	3.20	100	15.22	3.48	69	15.58	3.43	81	15.94	3.12	−0.25 (−1.12 to 0.62), − 0.08 (− 0.37 to 0.20)	0.54 (− 0.40 to 1.49), 0.15 (− 0.17 to 0.48)
Adolescent intended help-seeking from parent	140	2.95	1.21	158	2.84	1.29	86	2.95	1.21	100	2.87	1.29	68	2.78	1.43	80	2.64	1.39	0.04 (−0.35 to 0.43), 0.06 (− 0.23 to 0.35)	−0.11 (− 0.54 to 0.32), − 0.15 (− 0.48 to 0.18)
	n	%		n	%		n	%		n	%		n	%		n	%		OR (95% CI)	OR (95% CI)
Weak not sick stigma (parent) – median or below																			0.94 (0.34 to 2.60)	0.59 (0.19 to 1.80)
K6 above cut-off (parent)	28	18.5		37	21.9		14	14.6		17	15.6		10	12.5		19	19.4		0.81 (0.20 to 3.28)	1.68 (0.38 to 7.48)
K6 above cut-off (adolescent)	33	23.4		42	26.6		15	17.4		36	36.0		22	32.8		26	32.1		4.00 (1.09 to 14.71)^*^	0.62 (0.16 to 2.34)
Parent sought appropriate help for own mental health problem	36	81.8		49	69.0		16	72.7		23	69.7		10	58.8		28	66.7		42.0 (0.38 to 4622.42)	189.52 (0.61 to 58,672.80)
Adolescent sought help from parent for mental health problem	22	75.9		24	77.4		6	60.0		11	50.0		6	54.6		14	66.7		0.56 (0.01 to 26.25)	0.49 (0.01 to 28.48)
Adolescent sought help from health professional for mental health problem	19	65.5		23	74.2		8	80.0		12	54.6		4	36.4		16	76.2		0.21 (0.02 to 1.83)	4.52 (0.57 to 35.75)

*p < .05, **p < .01, ***p < .001

# Positive Cohen’s d favours MHFA and negative d favours PFA

M = mean, SD = standard deviation

## Discussion

This study aimed to test whether Youth Mental Health First Aid training received by parents of adolescents led to more effective support towards their adolescent and improved adolescent mental health outcomes. Our results showed no difference between MHFA training and physical first aid training in the quality of support parents provided to adolescents with a mental health problem, irrespective of whether this was assessed from the perspective of the adolescent or parent. Similarly, there was no evidence that Youth MHFA training benefited adolescent mental health as assessed by the SDQ. Secondary outcomes showed a mixed pattern of results, with some outcomes showing benefits, consistent with prior evaluations of MHFA training [9]. These effect sizes were generally somewhat smaller than previously found at up to 6 months after training, which is to be expected given the longer follow-up intervals in this study.

A key limitation of this study was a lack of power to detect primary outcome effects. The study did not reach its recruitment target of 990 families and faced greater attrition rates than expected. This meant that the number of adolescents with a mental health problem at follow-up was well below the required 128 to detect a medium effect size, which complicates interpretation of the results. In fact, effects were very small for the quality of mental health first aid that parents provided to adolescents, but effects were in the wrong direction for adolescent perceptions of parental support at 1-year follow-up. The main difficulties in recruiting were because parents found it difficult to commit to a 2-day course or wanted to be able to choose their preferred course type. Other constraints included course cancellations due to low numbers, or parents wishing to reschedule their course date because it no longer suited them. These recruitment difficulties reflect the broader challenge of engaging parents of adolescents in face-to-face group programs associated with their child’s mental health [21]. Barriers to attending a 2-day training course may have affected some parents disproportionately, such as those who had to travel a greater distance to attend training and for whom time and financial barriers reduced engagement, those who work long hours, or those who do not have access to childcare that would facilitate attendance. A future initiative at MHFA Australia will be a blended eLearning model with only 7 h of face-to-face content, which may reduce these barriers and attract parents in larger numbers.

An alternative interpretation of the pattern of results is that, despite the training improving parents’ knowledge about youth mental health problems and confidence and intentions to help, this did not translate into better support provided to adolescents. Previous research has shown that mental health first aid intentions positively predict behaviours at follow-up (r = 0.27) [22]. This study found the same strength of association between baseline intentions and support provided at 2-year follow-up (r = 0.27), but no association between baseline and 1-year follow-up (r = − 0.04). It is acknowledged that there are other factors important in the link between intentions and behaviour beyond having sufficient knowledge and confidence in what to do. Qualitative research on the process of providing mental health first aid shows that many factors influence the decision to provide help and the best help to offer, including the relationship between helper and recipient, the helper’s feelings of responsibility, personality, experience and values, and the recipient’s readiness for help [23]. Factors such as relationship quality could be measured in future studies of MHFA to determine whether they moderate the provision of support. In addition, further qualitative research to understand what barriers may prevent parents from implementing MHFA with their children would be useful. These may be important to target in future revisions to course content.

While parents are a target for training in Youth MHFA, most courses are delivered to professional groups. Training is frequently run for high school staff, including teachers, administrators and ancillary staff. An advantage of this model is that school staff have contact with many young people each year, can support each other to provide mental health first aid, and can seek the assistance of another school staff member if specific interpersonal barriers exist between themselves and the student. The presence of family conflict (which may arise as a by-product of a mental health problem) could make it difficult for a parent to assist their child. In addition, parents may only have contact with one adolescent that is both regular enough and intense enough to recognise a problem, whereas school staff may be in a better position to recognise patterns of behaviour, having a larger sample of adolescents to observe over time. The training is also run in other community settings such as sports clubs. Adults in these settings may have less formal relationships with the young people they interact with, making them another good target. Like school staff, they are likely to encounter many adolescents each year, increasing the likelihood of having the opportunity to offer mental health first aid.

This study has several strengths. It was rigorously designed with an active control condition and a long period of follow-up for a mental health education course. Furthermore, the impact of training was targeted towards a key period of life when the first-onset of mental disorders is high and early intervention would be most beneficial. Including parent and adolescent dyads also allowed for the direct measurement of MHFA effects on the recipient of aid, rather than relying solely on indirect reports from first aiders. As stated above, the main limitation was being underpowered, but additionally the primary outcome of adolescent perception of parental support for their mental health problem was developed for this study and has not been validated. Using a single-item with a limited response scale may not have been sensitive enough to the effects of MHFA training. Future research could explore a more nuanced or objective measure suitable for use with young adolescents.

## Conclusions

Ultimately, the question of whether MHFA training leads to better outcomes in the recipients of aid remains to be further explored. Although approaching a person, listening to their concerns and providing them with emotional support is perceived to be helpful [24], the full benefits from providing mental health first aid may only be realised when the recipient obtains professional help and receives an adequate course of treatment. It is known that many people do not receive a minimum dose of an evidence-based treatment when receiving mental health care, which remains a broader challenge to solve [25].

## Additional file

Additional file 1: **Table S1.** Vignettes depicting teenagers with a mental health problem. (DOCX 15 kb)

## Abbreviations

EDNOS: Eating Disorder Not Otherwise Specified
MHFA: Mental Health First Aid
OSFED: Other Specified Feeding and Eating Disorder
PFA: Provide First Aid
SDQ: Strengths and Difficulties Questionnaire
YMHFA: Youth Mental Health First Aid
