# Supplementary material for: Does Mental Health First Aid training improve the mental health of aid recipients? The training for parents of teenagers randomised controlled trial
Source: BMC Psychiatry. 2019 Mar 27;19:99. doi: 10.1186/s12888-019-2085-8 (PMC6437895; doi:10.1186/s12888-019-2085-8)
Supplement: Supplementary file 1 — Table S1. Vignettes depicting teenagers with a mental health problem. (DOCX 15 kb) [file 12888_2019_2085_MOESM1_ESM.docx]

Additional file 1

Table S1. Vignettes depicting teenagers with a mental health problem

| Depression (presented as Justin or Justine) | Justin is a 15 year old who has been feeling unusually sad and miserable for the last few weeks. He is tired all the time and has trouble sleeping at night. Justin doesn’t feel like eating and has lost weight. He can’t keep his mind on his studies and his marks have dropped. He puts off making any decisions and even day-to-day tasks seem too much for him. His parents and friends are very concerned about him. |
| --- | --- |
| Social phobia (presented as Paul or Paula) | Paul is a 15 year old living at home with his parents. Since starting his new school last year he has become even more shy than usual and has made only one friend. He would really like to make more friends but is scared that he’ll do or say something embarrassing when he’s around others. Although Paul’s work is OK he rarely says a word in class and becomes incredibly nervous, trembles, blushes and seems like he might vomit if he has to answer a question or speak in front of the class. At home, Paul is quite talkative with his family, but becomes quiet if anyone he doesn’t know well comes over. He never answers the phone and he refuses to attend social gatherings. He knows his fears are unreasonable but he can’t seem to control them and this really upsets him. |
| Eating Disorder (NOS) (presented as Simon or Simone) | Simon is a 15 year old living at home with his parents. A few times a week, when he is feeling upset, he sneaks food into his bedroom, including biscuits and chocolate bars. He eats until uncomfortably full, then feels guilty about overeating. Although he feels disgusted with himself about the amount he can eat, he cannot seem to stop himself from eating. When he is finished, he hides the empty plates and wrappers under the bed or under piles of clothes on the floor. The next day he tells himself that he will exercise hard to make up for it, running and doing sit-ups until he is exhausted. Simon’s mum has noticed that he has been doing this for at least half a year. |
| Psychosis (presented as John or Jenny) | John is a 15 year old who lives at home with his parents. He has been attending school irregularly over the past year and has recently stopped attending altogether. Over the past six months he has stopped seeing his friends and begun locking himself in his bedroom and refusing to eat with the family or to have a bath. His parents also hear him walking about in his bedroom at night while they are in bed. Even though they know he is alone, they have heard him shouting and arguing as if someone else is there. When they try to encourage him to do more things, he whispers that he won’t leave home because he is being spied upon by the neighbour. They realize he is not taking drugs because he never sees anyone or goes anywhere. |
